# Short-Term Intensive Insulin Therapy as a Remission Induction Strategy in Newly Diagnosed Type 2 Diabetes Mellitus: A Systematic Review

**DOI:** 10.7759/cureus.113192

**Published:** 2026-07-22

**Authors:** Tahira Nasreen, Noor ul huda Awan, Mohammad Uzair, Sunita Kumawat, FNU Vanshika, Esha Sharma, Daniel E Cook, Ghazanfar Ali, Ahmad Sattar

**Affiliations:** 1 Obstetrics and Gynaecology, Worcestershire Acute Hospitals NHS Trust, Worcester, GBR; 2 Internal Medicine, Rawalpindi Medical University, Rawalpindi, PAK; 3 Internal Medicine, Belarusian State Medical University, Minsk, BLR; 4 Internal Medicine, Prime West Consortium (PWC)/St. Francis Medical Center, Lynwood, USA; 5 Internal Medicine, Peoples University of Medical and Health Sciences, Hyderabad, PAK; 6 Internal Medicine, Wroclaw Medical University, Wroclaw, POL; 7 Internal Medicine, Avalon University School of Medicine, Youngstown, USA; 8 Internal Medicine, Medicare Hospital, Faisalabad, PAK; 9 Internal Medicine, Allama Iqbal Medical College, Lahore, PAK

**Keywords:** beta cell function, continuous subcutaneous insulin infusion, glucotoxicity, glycemic remission, insulin sensitivity, short term intensive insulin therapy, type 2 diabetes mellitus

## Abstract

Type 2 diabetes mellitus (T2DM) is characterized by progressive β-cell dysfunction, insulin resistance, and glucotoxicity, some of which may remain reversible early in the disease course. Short-term intensive insulin therapy (SIIT) may rapidly normalize hyperglycemia and facilitate β-cell recovery in newly diagnosed T2DM. This systematic review evaluated the effects of SIIT on glycemic control, drug-free remission, β-cell function, durability, and safety. PubMed/MEDLINE, Scopus, and Web of Science were searched for English-language randomized controlled trials published during the five-year period ending in April 2026. Four trials met the eligibility criteria. Because interventions, remission definitions, maintenance strategies, and follow-up periods differed substantially, study-level effect estimates, 95% confidence intervals, and p values were synthesized without statistical pooling. SIIT consistently improved early glycemic control, β-cell function, and insulin sensitivity. In one 245-participant trial, medication-supported hemoglobin A1c (HbA1c) below 6.5% at three months was achieved by 78.7% of participants receiving SIIT plus metformin-pioglitazone versus 59.0% receiving SIIT alone (adjusted p < 0.05); however, 12-month drug-free remission rates were similar at 50.0% and 50.6%, respectively (p = 0.972). Another trial found that linagliptin-metformin maintenance after SIIT increased the adjusted odds of achieving HbA1c below 7.0% at 48 weeks (odds ratio 2.78, 95% confidence interval 1.37-5.65; p = 0.005), although this represented medication-supported glycemic control rather than remission. In a two-year trial, induction SIIT improved β-cell function and insulin sensitivity (all p ≤ 0.0004), but repeated intermittent SIIT did not improve the Insulin Secretion-Sensitivity Index-2 (adjusted difference −35, 95% confidence interval −66 to −3; p = 0.03). Severe hypoglycemia was not reported in trials providing detailed safety data, and transient weight gain was not consistently demonstrated. SIIT may therefore serve as a selective metabolic induction strategy in early T2DM, but durable remission remains uncertain and may depend on effective sequential treatment. Standardized remission definitions, validated response predictors, and longer comparative trials are required before routine implementation.

## Introduction and background

Type 2 diabetes mellitus (T2DM) is a progressive metabolic disorder characterized by varying degrees of insulin resistance, impaired insulin secretion, and progressive β-cell dysfunction [1]. Although conventional treatment strategies have traditionally emphasized stepwise pharmacologic intensification beginning with oral antihyperglycemic agents, increasing attention has been directed toward the potential reversibility of early metabolic dysfunction during the initial stages of disease development. Chronic hyperglycemia contributes to glucotoxic stress, oxidative injury, impaired first-phase insulin secretion, and worsening hepatic insulin resistance, all of which may accelerate β-cell exhaustion over time [2]. Evidence from experimental and clinical studies suggests that a proportion of β-cell dysfunction in newly diagnosed T2DM may remain functionally reversible if metabolic stress is rapidly corrected before irreversible cellular decline becomes established [3].

Short-term intensive insulin therapy (SIIT), commonly delivered through continuous subcutaneous insulin infusion (CSII) or multiple daily injection protocols, has emerged as a potential strategy for achieving rapid glycemic normalization in patients with newly diagnosed T2DM, particularly those presenting with severe hyperglycemia [4]. Beyond its immediate glucose-lowering effects, SIIT has been proposed to reduce glucotoxic burden, improve insulin sensitivity, and temporarily “unload” pancreatic β-cells, thereby facilitating partial restoration of endogenous insulin secretory capacity. Several randomized studies have demonstrated improvements in glycemic control, β-cell function indices, and short-term remission rates following SIIT-based interventions [5]. However, the extent to which these metabolic improvements translate into sustained long-term remission remains uncertain, with variability observed across patient populations, maintenance therapies, and follow-up durations.

More recently, the concept of remission-oriented diabetes management has gained increasing clinical and scientific interest, shifting attention toward early disease modification rather than exclusive long-term pharmacologic escalation. Within this framework, SIIT has been explored not only as a temporary glucose-lowering intervention but also as a potential metabolic induction strategy during periods of marked but potentially reversible glucotoxicity [6,7]. Nevertheless, important uncertainties remain regarding optimal patient selection, remission durability, maintenance treatment requirements, and the relationship between early β-cell recovery and long-term disease trajectory. In addition, heterogeneity in remission definitions and post-induction therapeutic strategies has complicated direct comparisons across studies [8].

Previous reviews have suggested that SIIT may improve glycemic control and β-cell function in newly diagnosed T2DM; however, much of this evidence predates recently published randomized controlled trials evaluating newer induction and post-induction treatment strategies. Important uncertainties also remain regarding the durability of remission, the contribution of maintenance pharmacotherapy, and the clinical characteristics associated with sustained response. An updated synthesis is therefore needed to integrate these recent trials and clarify whether early metabolic improvements translate into durable remission. Accordingly, this systematic review aims to evaluate the effects of SIIT on glycemic remission, β-cell functional recovery, and metabolic outcomes in adults with newly diagnosed or early T2DM. It further examines remission durability and synthesizes current evidence regarding the potential role of SIIT as a phenotype-directed induction strategy in early T2DM management.

## Review

Materials and methods

Study Design and Reporting Framework

This systematic review evaluated the effects of SIIT on glycemic remission, β-cell functional recovery, and metabolic outcomes in adults with newly diagnosed or early T2DM. The methodology was developed in accordance with the Preferred Reporting Items for Systematic Reviews and Meta-Analyses (PRISMA) 2020 guidelines [9]. The review question was structured according to the Population, Intervention, Comparator, and Outcomes (PICO) framework [10]. The protocol was not prospectively registered in PROSPERO or another public registry. The target population included adults with newly diagnosed or early T2DM. The intervention of interest was SIIT delivered through CSII or multiple daily injection protocols. Comparators included conventional oral antihyperglycemic therapy, lifestyle modification, metformin-based maintenance therapy, or alternative post-induction treatment strategies. Primary outcomes included glycemic remission, β-cell functional recovery, and long-term glycemic control. Two reviewers independently screened titles and abstracts and subsequently assessed potentially eligible full-text articles. Disagreements at either stage were resolved through discussion and consensus, with consultation of a third reviewer when required. Formal inter-reviewer agreement statistics were not calculated, but consensus was reached for all final eligibility decisions.

Literature Search Strategy

A structured literature search was conducted in PubMed/MEDLINE, Scopus, and Web of Science to identify randomized controlled trials evaluating SIIT in adults with newly diagnosed or early T2DM. The search was limited to studies published in English within the preceding five years, and the final search was completed in April 2026. The search strategy combined disease-related, intervention-related, and outcome-related terms using Boolean operators. Reference lists of eligible studies were also screened manually. The complete search strategy is summarized in Table 1.

**Table 1:** Search strategy used to identify eligible studies. CSII, continuous subcutaneous insulin infusion.

Database/Source	Search Terms Used	Filters/Restrictions	Additional Screening
PubMed/MEDLINE	“type 2 diabetes mellitus” OR “newly diagnosed type 2 diabetes” OR “early type 2 diabetes” AND “short term intensive insulin therapy” OR “intensive insulin therapy” OR “continuous subcutaneous insulin infusion” OR “CSII” OR “early insulin therapy” AND “glycemic remission” OR “beta cell function” OR “C-peptide” OR “glucotoxicity”	English language; adults; randomized controlled trials; studies from the preceding five years	Reference lists of eligible studies were manually screened
Scopus	Same core search terms adapted to database syntax	English language; adults; randomized controlled trials; studies from the preceding five years	Reference lists of eligible studies were manually screened
Web of Science	Same core search terms adapted to database syntax	English language; adults; randomized controlled trials; studies from the preceding five years	Reference lists of eligible studies were manually screened

Eligibility Criteria

Studies were selected using predefined eligibility criteria to maintain a focused review of SIIT as an induction strategy in newly diagnosed or early T2DM. Eligible studies were randomized controlled trials involving adult patients and reporting clinically relevant outcomes such as glycemic remission, β-cell function, insulin sensitivity, or glycemic control. The full inclusion and exclusion criteria are summarized in Table 2.

**Table 2:** Inclusion and exclusion criteria used for study selection.

Category	Inclusion Criteria	Exclusion Criteria
Population	Adults with newly diagnosed or early type 2 diabetes mellitus	Longstanding diabetes; type 1 diabetes mellitus; gestational diabetes; pediatric populations
Study design	Randomized controlled trials	Narrative reviews; observational studies; conference abstracts; animal studies; editorials
Intervention	Short-term intensive insulin therapy delivered through insulin infusion or intensive insulin regimens	Stem cell therapies; isolated incretin physiology studies; non-insulin-centered interventions
Comparator	Oral antihyperglycemic therapy, maintenance pharmacologic strategies, lifestyle modification, or alternative insulin-based protocols	Studies without a relevant comparator or intervention framework
Outcomes	Glycemic remission, β-cell function, insulin sensitivity, or glycemic control	Studies lacking clinically relevant metabolic or remission outcomes

Study Selection and Data Extraction

All retrieved records were screened independently by title and abstract. Full texts of potentially eligible studies were then assessed according to the predefined eligibility criteria. Extracted data included study design, population characteristics, baseline glycemic severity, SIIT protocol characteristics, comparator interventions, follow-up duration, remission definitions, β-cell function indices, and major metabolic outcomes. Particular attention was given to markers of β-cell recovery, including C-peptide responses, insulin secretion indices, and measures of glycemic durability.

Risk of Bias Assessment

The methodological quality of the included randomized controlled trials was assessed using the Cochrane Risk of Bias 2 (RoB 2) tool [11]. The evaluated domains included the randomization process, deviations from intended interventions, missing outcome data, outcome measurement, and selective reporting. Overall risk-of-bias judgments were categorized as low risk, some concerns, or high risk according to standard RoB 2 guidance. Given the open-label design and heterogeneity of maintenance treatment protocols across several included studies, particular attention was directed toward potential performance and reporting biases during evidence interpretation.

Statistical and Evidence Synthesis

Given the limited number of eligible studies and substantial clinical and methodological heterogeneity in SIIT protocols, comparator interventions, remission definitions, maintenance strategies, follow-up durations, and reported endpoints, statistical pooling was considered inappropriate. A structured quantitative narrative synthesis was therefore performed. For each study, dichotomous outcomes were summarized using event counts, proportions, and study-reported effect estimates with 95% confidence intervals and P values, while continuous outcomes were presented using means, changes from baseline, adjusted between-group differences, 95% confidence intervals, and P values where available. Drug-free remission was distinguished from medication-supported achievement of glycemic targets. Findings were organized according to remission and glycemic control, β-cell function and insulin sensitivity, predictors of response, remission durability or relapse, and safety outcomes, including hypoglycemia and body-weight changes. Predictors were reported only when evaluated within the included studies, and no new causal inferences were made from unadjusted associations. Because the outcomes were not sufficiently comparable, no pooled effect estimate or quantitative heterogeneity statistic was calculated.

Study Selection Process

**Figure 1:**
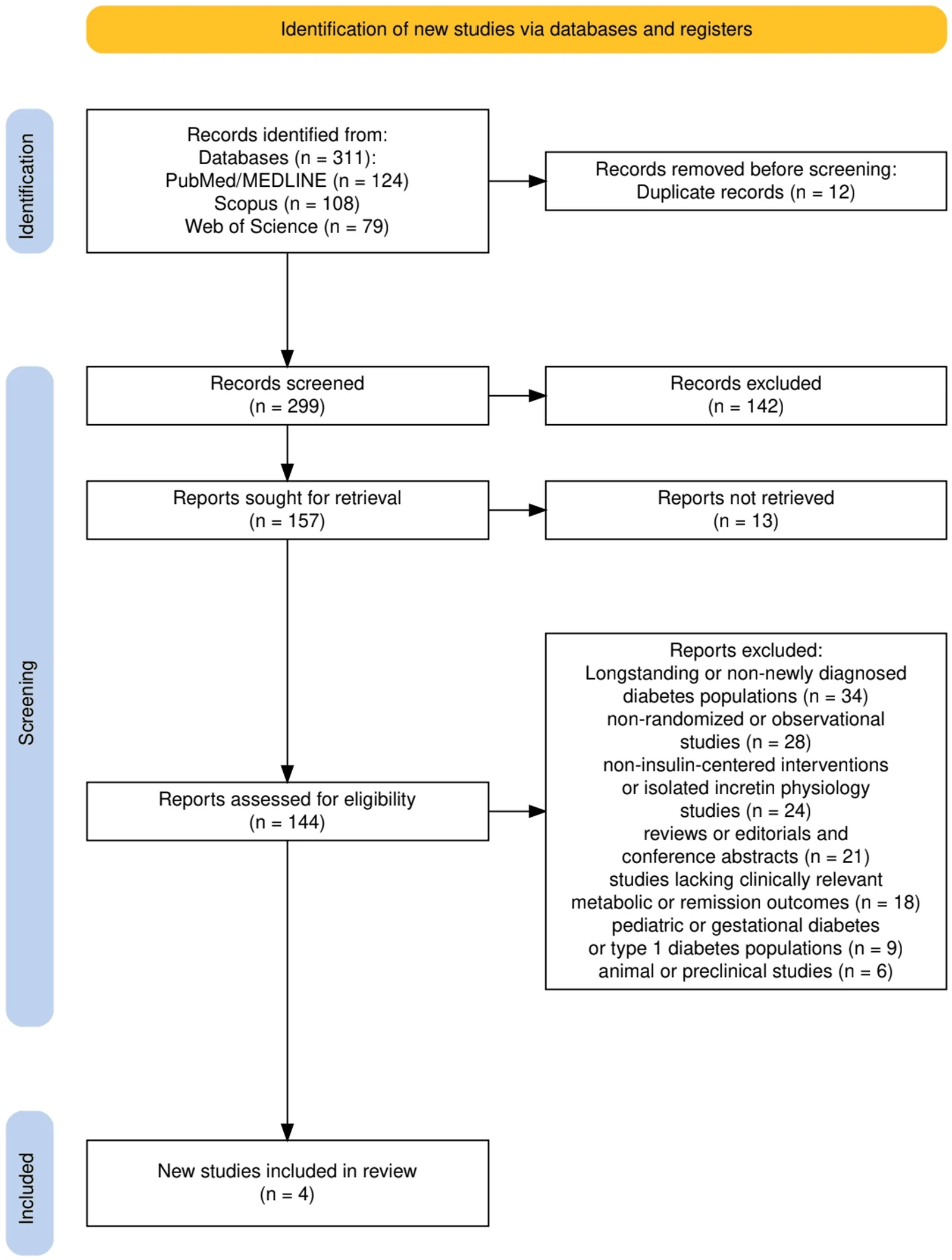
Preferred Reporting Items for Systematic Reviews and Meta-Analyses (PRISMA) flow diagram illustrating the study identification, screening, eligibility assessment, and inclusion process for studies evaluating short-term intensive insulin therapy in newly diagnosed type 2 diabetes mellitus.

The study selection process is summarized in Figure 1. A total of 311 records were identified through database searching, including PubMed/MEDLINE (n = 124), Scopus (n = 108), and Web of Science (n = 79). After removal of 12 duplicate records, 299 studies underwent title and abstract screening, of which 142 were excluded according to the predefined eligibility criteria. Full texts of 157 reports were sought for retrieval, and 13 reports were unavailable for assessment. The remaining 144 full-text articles were evaluated for eligibility. The most common reasons for exclusion were longstanding or non-newly diagnosed diabetes populations, non-randomized or observational designs, non-insulin-centered interventions, and absence of clinically relevant metabolic or remission outcomes. Following full-text review, four randomized controlled trials met the final inclusion criteria and were included in the synthesis. The detailed PRISMA flow diagram outlining study identification, screening, eligibility assessment, and inclusion is presented in Figure 1.

Results

Characteristics of the Selected Studies

The characteristics of the four included randomized controlled trials are summarized in Table 3. Participants had newly diagnosed or early T2DM, although baseline glycemic severity varied across studies. SIIT was delivered through CSII or short-duration intensive injection regimens, followed by lifestyle management, metformin, dipeptidyl peptidase-4 inhibitors, insulin sensitizers, or intermittent repeat insulin therapy. Follow-up ranged from 48 weeks to two years. Liu et al. [12] assessed medication-supported hemoglobin A1c (HbA1c) target attainment, Ke et al. [13] formally evaluated drug-free remission, Stojanovic et al. [14] examined glycemic control and stimulated C-peptide responses, and Retnakaran et al. [15] primarily evaluated β-cell preservation during metformin maintenance. Differences in outcome definitions, maintenance strategies, and follow-up precluded direct comparison of all endpoints.

**Table 3:** Characteristics and major findings of randomized controlled trials evaluating short term intensive insulin therapy in newly diagnosed or early type 2 diabetes mellitus. SIIT, short term intensive insulin therapy; T2DM, type 2 diabetes mellitus; HbA1c, hemoglobin A1c; CSII, continuous subcutaneous insulin infusion; AIR, acute insulin response; IIT, intensive insulin therapy; ISSI-2, Insulin Secretion Sensitivity Index-2.

Study	Population	Intervention (SIIT Strategy)	Comparator	Follow-up	Primary Outcomes	Key Findings
Liu et al., 2024 [12]	Newly diagnosed T2DM with HbA1c ≥8.5%	2–3 weeks SIIT followed by linagliptin, metformin, or combination therapy	Lifestyle modification alone after SIIT	48 weeks	HbA1c <7.0%, β-cell function	Post-SIIT oral therapy, especially linagliptin + metformin, improved sustained glycemic control and β-cell recovery
Ke et al., 2026 [13]	Newly diagnosed T2DM	2-week CSII-based SIIT alone or combined with metformin/pioglitazone or sitagliptin	CSII alone	12 months	Diabetes remission, β-cell function, glycemic control	Combination therapy improved short-term glycemic outcomes and AIR, but 12-month remission rates were similar between groups
Stojanovic et al., 2023 [14]	Newly diagnosed T2DM with HbA1c ≥9%	One-month early insulin therapy + metformin	Glimepiride + metformin	12 months	β-cell function, HbA1c, lipid control	Early insulin produced greater and more sustained β-cell recovery and glycemic improvement than oral therapy
Retnakaran et al., 2021 (RESET-IT Main) [15]	Early T2DM (median duration 1.3 years)	3-week induction IIT followed by metformin ± periodic 2-week IIT every 3 months	Metformin alone after induction IIT	2 years	β-cell function (ISSI-2), HbA1c	Initial IIT improved metabolic parameters, but intermittent repeat IIT did not further enhance long-term β-cell preservation

Quality Assessment

The risk of bias assessment of the included randomized controlled trials is presented in Table 4. Overall, the methodological quality of the included studies was considered acceptable, although most trials demonstrated some concerns in specific domains. The primary sources of potential bias were related to open-label study designs and heterogeneity in post-intervention maintenance strategies, which may have influenced adherence patterns and subsequent metabolic outcomes. Nevertheless, objective outcome measures such as HbA1c, β-cell function indices, C-peptide responses, and insulin sensitivity assessments reduced the likelihood of substantial measurement bias across studies. Randomization processes and outcome reporting were generally adequate, with no study demonstrating a consistently high risk of bias across multiple domains. Collectively, the included evidence was considered sufficiently robust to support synthesis while acknowledging the methodological limitations inherent to the currently available literature.

**Table 4:** Risk of bias assessment of included randomized controlled trials using the Cochrane Risk of Bias 2 tool. RoB 2, Risk of Bias 2 [11]; HbA1c, hemoglobin A1c; ISSI-2, Insulin Secretion Sensitivity Index-2; OGTT, oral glucose tolerance test.

Study	Suitable Risk-of-Bias Tool	Randomization Process	Deviations From Intended Intervention	Missing Outcome Data	Outcome Measurement	Selective Reporting	Overall Risk of Bias
Liu et al., 2024 [12]	Cochrane RoB 2	Low concern	Some concern: open-label design	Low concern	Some concern: glycemic outcomes mostly objective, but open-label follow-up may influence management	Low concern	Some concerns
Ke et al., 2026 [13]	Cochrane RoB 2	Low concern	Some concern: open-label, multiple active treatment arms	Low concern	Some concern: remission and HbA1c are objective, but treatment awareness may affect adherence/management	Low concern	Some concerns
Stojanovic et al., 2023 [14]	Cochrane RoB 2	Some concern: smaller single-trial design, randomization details need confirmation	Some concern: open-label treatment likely	Low/some concern	Low concern: HbA1c and C-peptide are objective	Some concern: protocol/registration should be checked	Some concerns
Retnakaran et al., 2021 (RESET-IT Main) [15]	Cochrane RoB 2	Low concern	Some concern: intervention assignment likely not blinded	Low concern	Low concern: ISSI-2, OGTT, HbA1c are objective	Low concern	Some concerns

Glycemic Control and Remission Outcomes

In Liu et al., 2024 [12], medication-supported HbA1c below 6.5% at 48 weeks was achieved by 70%, 68%, and 68% of participants receiving linagliptin plus metformin, linagliptin, and metformin after SIIT, respectively, compared with 48% receiving no maintenance medication (overall p = 0.005). Linagliptin plus metformin increased the adjusted odds of achieving HbA1c below 7.0% versus control (OR 2.78, 95% CI 1.37-5.65; p = 0.005). These outcomes represented maintained glycemic control rather than drug-free remission. In Ke et al., 2026 [13], HbA1c below 6.5% at three months was achieved by 78.7% with SIIT plus metformin and pioglitazone, 65.9% with SIIT plus sitagliptin, and 59.0% with SIIT alone (overall p = 0.031). However, 12-month drug-free remission rates were similar at 50.0%, 48.8%, and 50.6%, respectively (p = 0.972). Stojanovic et al., 2023 [14] reported lower HbA1c with early insulin plus metformin than with glimepiride plus metformin at three months (6.26% ± 0.18% vs. 6.78% ± 0.10%; p = 0.016), although the between-group difference was not significant at 12 months (p = 0.056). In Retnakaran et al., 2021 (RESET-IT Main) [15], two-year HbA1c was comparable between metformin plus intermittent intensive insulin therapy and metformin alone (6.4% ± 0.1% vs. 6.3% ± 0.1%; p = 0.46), with 32.6% of participants in each group maintaining HbA1c of 6.0% or lower.

β-Cell Function and Insulin Sensitivity

In Liu et al., 2024 [12], the 48-week Insulin Secretion-Sensitivity Index-2 was higher with linagliptin plus metformin than with no maintenance medication (345.7, 95% CI 310.4-379.0 vs. 265.4, 95% CI 231.4-299.5; p = 0.009). Between-group differences in HOMA-IR and the Matsuda index were observed overall (p = 0.02 and p = 0.03, respectively), but individual comparisons were not significant after adjustment for multiple testing. In Ke et al., 2026, [13] both combination regimens produced greater acute insulin responses than SIIT alone immediately after treatment (metformin-pioglitazone, p = 0.003; sitagliptin, p = 0.039). Metformin-pioglitazone also improved HOMA-β (p = 0.035) and the disposition index (p < 0.001), while lowering HOMA-IR relative to sitagliptin (p = 0.009); these differences were no longer significant at three months. Stojanovic et al., 2023 [14] found a greater BMI-adjusted stimulated C-peptide response with early insulin than with glimepiride at three months (4.60 ± 0.59 vs. 3.21 ± 0.34 m²/kg; p = 0.044), which persisted at 12 months (4.57 ± 0.56 vs. 3.04 ± 0.34 m²/kg; p = 0.023). In Retnakaran et al., 2021 (RESET-IT Main) [15], induction therapy improved β-cell function, whole-body insulin sensitivity, and hepatic insulin resistance (all p ≤ 0.0004); however, repeated intermittent insulin did not improve two-year ISSI-2 compared with metformin alone (adjusted difference −35, 95% CI −66 to −3).

Predictors of Successful Response

Formal predictor assessment was limited across the included trials. Stojanovic et al., 2023 [14] found that baseline C-peptide and stimulated C-peptide response were associated with BMI but not with baseline HbA1c or age; however, an adjusted remission-prediction model was not reported. Retnakaran et al., 2021 (RESET-IT Main) [15] adjusted the primary β-cell outcome for baseline ISSI-2 but did not validate disease duration, baseline β-cell reserve, early glycemic response, or insulin sensitivity as predictors of drug-free remission. The other trials primarily evaluated treatment effects rather than predictors. Consequently, predictor assessment was inconsistent, and no variable was validated as a reproducible predictor of drug-free remission across more than one study.

Remission Durability and Glycemic Relapse

Evidence of long-term durability was inconsistent. In Liu et al., 2024 [12], 41 participants required additional glucose-lowering therapy because of hyperglycemic relapse, with 73% of these events occurring within 24 weeks after SIIT. In Ke et al., 2026 [13], the three-month advantage in HbA1c target attainment with metformin and pioglitazone was not sustained after treatment withdrawal; 12-month remission rates were approximately 50% in all groups (p = 0.972). Stojanovic et al., 2023 [14] similarly found that the HbA1c advantage of early insulin was significant at three months (p = 0.016) but not at 12 months (p = 0.056). In Retnakaran et al., 2021 (RESET-IT Main) [15], adding two-week courses of intensive insulin every three months did not improve β-cell preservation or HbA1c at two years. Collectively, the trials demonstrated attenuation of early treatment effects over time, but differences in outcome definitions prevented calculation of a pooled remission-decay rate.

Safety and Body-Weight Outcomes

In Liu et al., 2024 [12], participants experienced a mean of 2.0 ± 2.6 level 1 and 0.15 ± 0.46 level 2 hypoglycemic episodes during SIIT, without significant between-group differences or severe hypoglycemia. Median body weight decreased by 2.0 kg (IQR -4.7 to 0.5), with no between-group difference (p = 0.23). Gastrointestinal adverse effects occurred in 17% receiving linagliptin plus metformin and 16% receiving metformin alone, causing eight treatment withdrawals. In Ke et al., 2026 [13], hypoglycemic episodes differed among the metformin-pioglitazone, sitagliptin, and SIIT-only groups (median 3 vs. 2 vs. 3 episodes; p = 0.005), with no severe episodes. Weight decreased in all groups at three months (−4.1 ± 2.4, −3.3 ± 2.9, and −2.6 ± 2.5 kg; p = 0.002), but differences were absent at 12 months (p = 0.738). Mild gastrointestinal symptoms were more frequent with metformin-pioglitazone than sitagliptin (16.8% vs. 3.7%; p = 0.004). Stojanovic et al., 2023 [14] reported no adverse effect on body weight, whereas detailed safety outcomes were not consistently reported by Retnakaran et al., 2021 (RESET-IT Main) [15]. Overall, transient weight gain was not demonstrated in the included trials, although safety and weight reporting was incomplete.

Quantitative Outcomes

Table 5 summarizes the principal inferential efficacy, durability, and safety findings. Outcomes are presented separately because differences in interventions and endpoint definitions precluded statistical pooling.

**Table 5:** Principal quantitative efficacy, durability, and safety outcomes of the included randomized trials. HbA1c, hemoglobin A1c; SIIT, short-term intensive insulin therapy; IIT, intensive insulin therapy; ISSI-2, Insulin Secretion-Sensitivity Index-2; AIR, acute insulin response; OR, odds ratio; CI, confidence interval.

Study	Principal glycemic or remission outcome	β-cell function and durability	Safety and weight
Liu et al., 2024 [12]	HbA1c <6.5% at 48 weeks: 70% with linagliptin–metformin vs. 48% with no maintenance therapy (p = 0.005). Adjusted OR for HbA1c <7.0%: 2.78 (95% CI 1.37–5.65; p = 0.005)	ISSI-2: 345.7 (95% CI 310.4-379.0) vs. 265.4 (95% CI 231.4-299.5; p = 0.009). Of 41 relapses, 73% occurred within 24 weeks	No severe hypoglycemia. Median weight change -2.0 kg; no between-group difference (p = 0.23)
Ke et al., 2026 [13]	Three-month HbA1c <6.5%: 78.7% with metformin-pioglitazone vs. 59.0% with SIIT alone (adjusted p < 0.05). Twelve-month remission: 50.0% vs. 50.6% (p = 0.972)	AIR was greater with metformin-pioglitazone than SIIT alone (86.60 vs. 53.70 μU·min/mL; p = 0.003), but the advantage was not sustained	Hypoglycemia differed among groups (p = 0.005), without severe events. Three-month weight change: -4.1 vs. -2.6 kg (p = 0.002)
Stojanovic et al., 2023 [14]	Three-month HbA1c: 6.26% with early insulin vs. 6.78% with glimepiride (p = 0.016); difference not significant at 12 months (p = 0.056)	Twelve-month BMI-adjusted stimulated C-peptide: 4.57 vs. 3.04 m²/kg (p = 0.023)	No adverse effect on body weight; detailed hypoglycemia rates were not reported
Retnakaran et al., 2021 (RESET-IT Main) [15]	Two-year HbA1c: 6.4% with intermittent IIT vs. 6.3% with metformin alone (p = 0.46); 32.6% in each group maintained HbA1c ≤6.0%	Two-year ISSI-2: adjusted difference -35 (95% CI -66 to -3; p = 0.03), showing no benefit from repeated IIT	Detailed hypoglycemia and weight outcomes were not consistently reported

Discussion

Opening Synthesis

The included randomized trials demonstrated that SIIT can rapidly improve glycemic control and markers of β-cell function in early or newly diagnosed T2DM. Liu et al. [12], Ke et al. [13], and Stojanovic et al. [14] reported early metabolic benefits following insulin-based induction, particularly in patients with marked hyperglycemia. Retnakaran et al. [15] similarly demonstrated improvements in β-cell function and insulin sensitivity after induction therapy, although repeated intermittent insulin courses did not provide additional long-term preservation. Importantly, medication-supported glycemic control should be distinguished from drug-free remission. Although early improvements were consistently observed, remission and glycemic durability varied according to maintenance strategy and follow-up duration. SIIT may therefore be best understood as a metabolic induction strategy that temporarily reverses glucotoxic dysfunction rather than as a standalone intervention that reliably produces sustained remission.

The Biology of Early Glucotoxicity Reversal

The rationale for SIIT is based on the potentially reversible component of glucotoxic β-cell dysfunction. Chronic hyperglycemia promotes oxidative stress, impairs first-phase insulin secretion, disrupts mitochondrial function, and aggravates hepatic and peripheral insulin resistance. Rapid glycemic normalization may interrupt this cycle, reduce β-cell secretory demand, and permit partial recovery of endogenous insulin responsiveness. Stojanovic et al. [14], Ke et al. [13], and Retnakaran et al. [15] demonstrated improvements in stimulated C-peptide responses, β-cell indices, or insulin sensitivity following SIIT. However, the subsequent attenuation of several treatment effects indicates that glucotoxicity reversal alone does not eliminate the underlying drivers of T2DM. SIIT may therefore create a temporary period of metabolic recovery during which effective maintenance treatment could help preserve β-cell function.

Why Early Disease Stage Matters

The potential benefit of SIIT appears most relevant during early T2DM, when β-cell dysfunction may remain partly reversible. Participants in the included trials generally had newly diagnosed or short-duration disease, and several cohorts presented with marked hyperglycemia, suggesting substantial glucotoxic stress without necessarily indicating irreversible β-cell depletion. Liu et al. [12] and Stojanovic et al. [14] demonstrated improved glycemic control and β-cell indices in patients with elevated baseline HbA1c, while Retnakaran et al. [15] showed improved insulin sensitivity and β-cell function after induction therapy in early disease. These findings support the biological importance of residual β-cell reserve, metabolic plasticity, and preserved hepatic insulin sensitivity. Nevertheless, these characteristics were not consistently evaluated in adjusted predictor models, and no baseline variable was validated across the included trials as a reproducible predictor of drug-free remission. Disease stage should therefore be viewed as a plausible treatment modifier rather than an established selection criterion.

Exploratory Predictors of Sustained Response

Although no predictor was validated across the four included trials, several factors may help explain variation in treatment response. Shorter disease duration and residual β-cell reserve are biologically plausible predictors because they may indicate a greater reversible component of β-cell dysfunction; however, neither was consistently tested as an independent predictor of drug-free remission. Early achievement of glycemic targets and improvement in β-cell function after SIIT may provide more informative indicators of metabolic reversibility. In a secondary analysis of the RESET-IT population, sustained β-cell stabilization occurred in 55 of 99 participants. After adjustment for age, diabetes duration, baseline β-cell function, and changes in adiposity, improvement in the Insulin Secretion-Sensitivity Index-2 during induction independently predicted stabilization (adjusted OR 1.02, 95% CI 1.00-1.03; p = 0.005), while worsening hepatic insulin resistance during maintenance reduced the likelihood of sustained response (adjusted OR 0.64, 95% CI 0.49-0.83; p = 0.0007) [16]. Weight and adiposity trajectories may influence durability through their effects on insulin sensitivity, although their independent predictive contribution remains uncertain. These findings are exploratory and relate to sustained β-cell stabilization rather than validated drug-free remission; therefore, they should not yet be used as definitive selection criteria for SIIT.

The Remission Durability Problem

Although SIIT consistently produced early metabolic improvement, these benefits frequently attenuated over time. In Ke et al. [13], three-month HbA1c target attainment was greater with metformin and pioglitazone than with SIIT alone, but 12-month drug-free remission rates were approximately 50% across all groups. Retnakaran et al. [15] likewise found that repeating intensive insulin courses every three months did not improve two-year β-cell preservation or glycemic control. In Liu et al. [12], 41 participants experienced hyperglycemic relapse requiring additional treatment, with 73% of relapses occurring within 24 weeks after SIIT. These findings suggest that SIIT can reverse acute glucotoxicity without fully modifying persistent insulin resistance, hepatic metabolic dysfunction, lipotoxicity, obesity-associated inflammation, or progressive β-cell attrition [17]. Incomplete lifestyle modification and heterogeneous maintenance strategies may further contribute to recurrent dysglycemia [18]. Thus, SIIT appears more effective for remission induction than for ensuring remission durability, which remains dependent on sustained control of the broader metabolic drivers of T2DM [19].

Why Combination Therapy May Matter

The contrasting findings of Liu et al. [12] and Ke et al. [13] suggest that the duration and continuity of treatment after SIIT may be as important as the induction phase. Liu et al. [12] found that continued oral therapy for 48 weeks, particularly linagliptin plus metformin, improved medication-supported glycemic control and β-cell function compared with no maintenance medication. In contrast, Ke et al. [13] showed that metformin-pioglitazone or sitagliptin improved early glycemic and β-cell outcomes, but these agents were discontinued after 90 days and did not increase 12-month remission. Metformin may help preserve insulin sensitivity, while dipeptidyl peptidase-4 (DPP-4) inhibition and insulin sensitization may reduce glycemic and secretory stress; however, the optimal agent, duration, and withdrawal strategy remain undefined. Furthermore, repeated insulin courses in Retnakaran et al. [15] did not compensate for the absence of a more effective maintenance approach. SIIT should therefore be considered an induction strategy whose long-term value may depend on an appropriately selected and sufficiently sustained sequential treatment regimen.

Safety Trade-offs: Hypoglycemia and Weight Change

Hypoglycemia and weight gain are important concerns when considering early insulin therapy. In Liu et al. [12], mild biochemical hypoglycemia occurred during SIIT, but no severe episodes were reported. Ke et al. [13] similarly found a between-group difference in hypoglycemic frequency (p = 0.005), with fewer episodes during sitagliptin-supported SIIT, although all events were corrected with carbohydrate intake and none were severe. These findings suggest that SIIT can be administered with an acceptable short-term safety profile when accompanied by frequent glucose monitoring and active insulin titration. Insulin-associated weight gain remains a recognized concern because correction of glycosuria and restoration of anabolic metabolism may increase energy retention. Nevertheless, weight gain was not demonstrated consistently in the included trials: Liu et al. [12] and Ke et al. [13] reported modest weight reductions, while Stojanovic et al. [14] observed no adverse effect on body weight. The short treatment periods and incomplete safety reporting, however, limit conclusions regarding longer-term weight effects. Because some sequential regimens included pioglitazone, future studies should also distinguish insulin-related adipose gain from thiazolidinedione-associated fluid retention. Monitoring for hypoglycemia, weight change, edema, and treatment burden should therefore remain integral to patient selection and follow-up.

Reconciling the Apparent Contradictions

The apparent discrepancy between improved β-cell function and inconsistent remission partly reflects differences in outcome definitions and trial design. Liu et al. [12] primarily evaluated HbA1c target attainment during maintenance pharmacotherapy, whereas Ke et al. [13] assessed drug-free remission after treatment withdrawal, and Retnakaran et al. [15] evaluated β-cell preservation during metformin maintenance. These endpoints are related but not interchangeable. Follow-up duration, baseline glycemic severity, SIIT protocols, and post-induction treatments also differed substantially [17]. Biologically, early β-cell recovery may be insufficient if insulin resistance, adiposity, hepatic dysfunction, and adverse metabolic behavior persist [19,20]. The evidence therefore supports a consistent early physiological response to SIIT but not a uniform probability of durable remission. Major sequential-treatment gaps remain, including the absence of standardized maintenance duration, withdrawal criteria, relapse thresholds, and comparative trials of newer glucose-lowering or weight-modifying therapies.

Clinical Implications

SIIT may be most relevant as a selective induction strategy for patients with newly diagnosed T2DM and marked symptomatic hyperglycemia or glucotoxicity rather than as universal first-line treatment. Liu et al. [12] and Stojanovic et al. [14] demonstrated meaningful glycemic and β-cell improvement in patients with high baseline HbA1c, supporting early correction of severe metabolic disturbance before β-cell dysfunction becomes less reversible. However, short disease duration, younger age, preserved β-cell reserve, and hepatic insulin sensitivity were not consistently validated as predictors of drug-free remission across the included trials. Patient selection should therefore consider hyperglycemic severity, hypoglycemia risk, ability to manage insulin, and the feasibility of subsequent maintenance therapy. Close glucose monitoring and dose titration are necessary during induction, although severe hypoglycemia was not reported in the trials providing detailed safety data. Current evidence supports SIIT as a potentially phenotype-directed induction strategy, but not as a curative or routinely applicable intervention [21].

Limitations

This review included only four randomized trials, limiting the precision and generalizability of its conclusions. Meta-analysis was not appropriate because SIIT protocols, comparators, remission definitions, maintenance therapies, follow-up durations, and metabolic endpoints differed substantially. A structured quantitative narrative synthesis was therefore used, with effect estimates, 95% confidence intervals, and p values reported when available. Several trials evaluated medication-supported HbA1c targets rather than drug-free remission, restricting direct comparison of remission rates. Predictor analyses were limited and inconsistent, while hypoglycemia, body-weight change, and other adverse events were not uniformly reported. Most studies were open-label, although their principal biochemical outcomes were objectively measured. Finally, follow-up of 48 weeks to two years was insufficient to establish long-term remission durability, β-cell preservation, or cardiometabolic benefit.

Future Research Directions

Several important uncertainties remain regarding the optimal clinical role of SIIT in early T2DM. Future studies should prioritize standardized definitions of diabetes remission to improve comparability across trials and facilitate clearer interpretation of long-term outcomes. Additional investigation is also needed to determine the optimal duration and intensity of SIIT protocols, particularly regarding the relative effectiveness of brief induction strategies versus prolonged or intermittent insulin courses. Given the variability in remission durability observed across studies, identifying predictors of sustained response, including residual β-cell reserve, hepatic insulin sensitivity, inflammatory burden, and metabolic phenotype, will be particularly important. Comparative studies evaluating continuous subcutaneous insulin infusion versus multiple daily injection approaches may further clarify whether specific delivery strategies influence metabolic recovery. Long-term cardiometabolic outcomes also remain insufficiently characterized, as most existing trials primarily focus on glycemic indices and β-cell function over relatively limited follow-up periods. Future research integrating cardiovascular, hepatic, and obesity-related endpoints may help determine whether early SIIT produces broader disease-modifying effects beyond short-term glycemic stabilization.

## Conclusions

Current evidence indicates that SIIT can produce rapid glycemic improvement and partial recovery of β-cell function and insulin sensitivity in selected patients with newly diagnosed T2DM, particularly those with marked hyperglycemia and potentially reversible glucotoxicity. However, medication-supported glycemic control should not be equated with drug-free remission, and early benefits are frequently attenuated after treatment withdrawal. Severe hypoglycemia was not reported in trials providing detailed safety data, while transient weight gain was not consistently demonstrated. Sustained benefit appears to depend on continued management of insulin resistance, adiposity, hepatic metabolic dysfunction, and progressive β-cell decline, yet the optimal post-SIIT treatment strategy remains undefined. Because the evidence comprises only four heterogeneous randomized trials with inconsistent remission definitions, limited predictor analyses, and follow-up of no more than two years, SIIT should be considered a promising induction strategy rather than a routine remission therapy. Larger multicenter trials using standardized remission criteria are needed to identify appropriate candidates and determine the optimal type and duration of sequential maintenance treatment.
